# A Rare Case of Survival after Complete Traumatic Cricotracheal Separation

**DOI:** 10.1155/2012/105763

**Published:** 2012-06-21

**Authors:** Gautam Bir Singh, Rajiv Kummar, Neha Verma, Anil K. Rai

**Affiliations:** ^1^Department of Otorhinolaryngology and Head-Neck Surgery, Lady Hardinge Medical College & Associated Hospitals, New Delhi 11000, India; ^2^Department of Otorhinolaryngology and Head-Neck Surgery, Vardhman Mahavir Medical College & Safdarjung Hospital, New Delhi 110029, India

## Abstract

We report the survival of an extremely rare case of complete cricotracheal transection following incisional trauma with knife. The clinical record underlines the importance of prompt airway management and immediate neck exploration in such cases. To the authors knowledge, such a case has not been reported previously in the literature.

## 1. Introduction

Cricotracheal separation of the upper airway following neck trauma is a rarity [1–3]. As very many patients do not survive this laryngotracheal injury [4–6], there is scant medical literature on the cited subject. With this background, the authors present one such rare case, where the mode of trauma was found to be interesting, hitherto unreported in medical literature. The case in focus also outlines the definitive management protocol for survival in this rare yet generally fatal laryngotracheal injury.

## 2. Case Report

A 19-year-old female reported to the casualty department of Vardhman Mahavir Medical College and Safdarjung hospital, New Delhi (a tertiary care university teaching hospital) with the history of cut throat injury occurring about 4 hours back. The patient was brought by the police, as it was referred from an adjoining district hospital with victim's action being reported as a suspected suicidal attempt. On examination, patients vitals were stable. A vertical wound of approximately 8 cm in linear length with clean margins and ooze of blood was seen almost in the middle of the neck. The injury site was having a tracheostomy tube of size: 8.5, through which the patient was maintaining adequate respiration. The referral record indicated that the upper airway had been totally transected and a tracheostomy tube had been inserted in the distal part of the trachea as a first aid measure in the district hospital. No bruises or any other lacerations were seen in relation to the said wound. Also, the hyoid and thyroid cartilage were clinically intact. 

**Figure 1:**
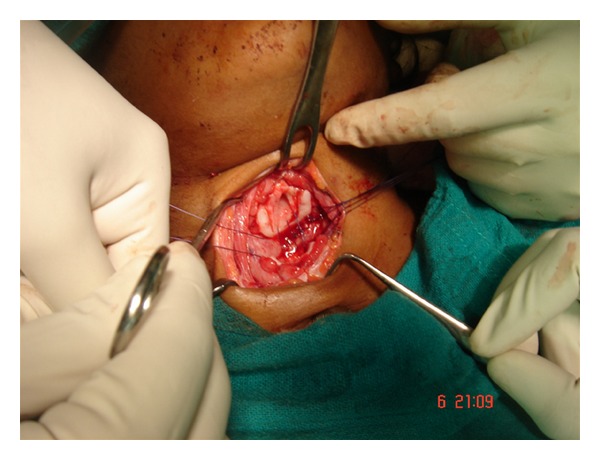
Clinical photograph showing cricotracheal separation.

**Figure 2:**
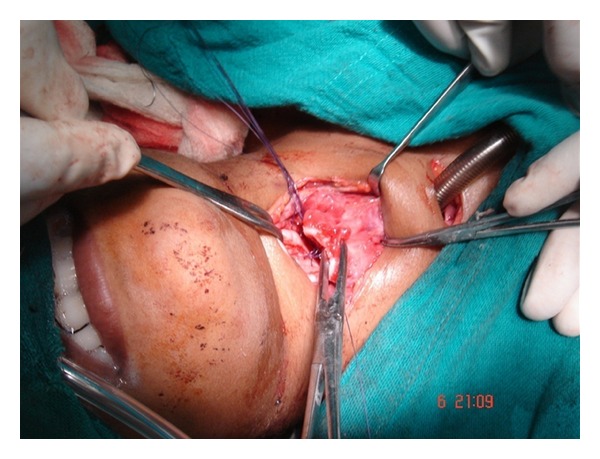
Clinical photograph showing end to end anastomosis with mobilization of trachea.

After orthopedic clearance for any suspected spinal trauma, the patient was transferred to ENT department and an immediate exploration of the injury under general anesthesia was undertaken. On exploration of the wound, complete cricotracheal transection with wide separation of the ends, anterior wall perforation of the esophagus, and cutting off the strap muscles was observed (Figure 1). No injury to great vessels or to any other adjoining structures in the neck was observed. A Ryle's tube was immediately inserted and the anterior wall of the esophagus was repaired surgically by suturing. The distal end of the trachea was retracted approximately 3 cm, the trachea was retrieved manually and primary end to end anastomosis of the tracheal injury was done (Figure 2). The recurrent laryngeal nerve on either side of neck was not looked for. Finally, wound was stitched in two layers with a simple corrugated drain in situ.

**Figure 3:**
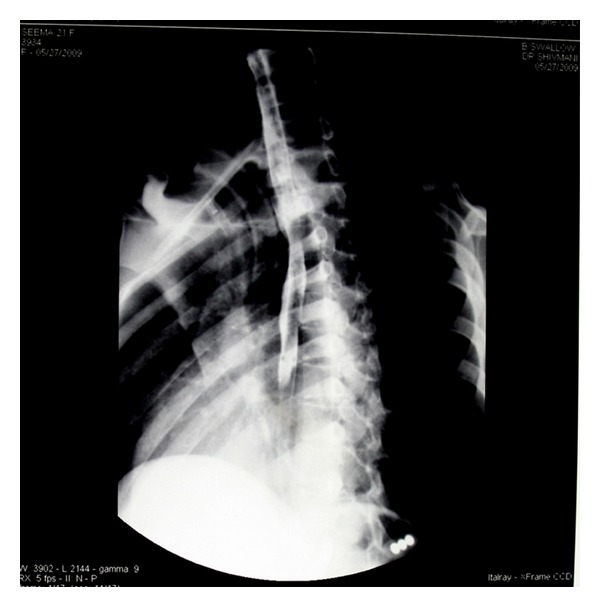
Barium swallow showing normal study.

**Figure 4:**
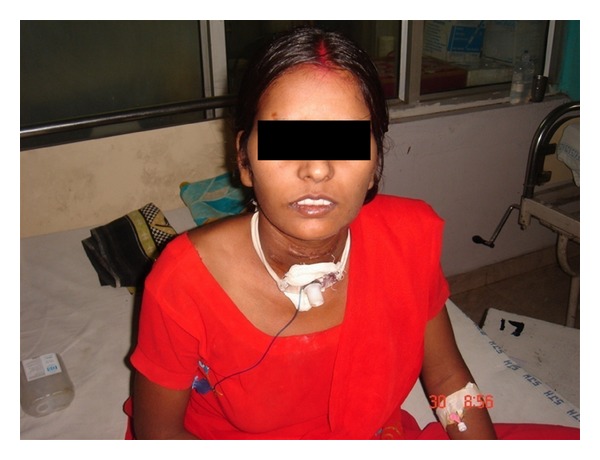
Photograph of the patient at the time of discharge.

Postoperatively, the patient was put on antibiotics and the drain was removed after 48 hours. The patient underwent CT-Scan (to document the injury for medico legal purposes) and fibro-optic laryngoscopy on the 7th postoperative day which revealed the vocal cords in paramedian position. A barium swallow was done at the end of 2nd week, which was found to be normal, thereafter feeding to the patient was started orally (Figure 3). A repeat bronchscopy was done at the end of third week which revealed no abnormality in the end to end anastomosis suture line, a repeat fibroptic laryngoscopy at the same time, however, once again revealed the cords in paramedian position: bilateral recurrent laryngeal nerve palsy. Subsequently, the patient was discharged after 3 weeks with tracheostomy tube in situ (Figure 4). The patient is in regular monthly followup with ENT department with no untoward incident to report. 

## 3. Discussion

Most of the rare cases of cricotracheal separation are defined with blunt trauma of the clothes line type injury-exposed larynx striking a fixed cable at high speed [7, 8]. There is a marked paucity of medical literature on cricotracheal transection due to deep incisional injury of neck. We thus describe our experience with one survivor of this injury due to deep incisional knife trauma.

The patient gave us an interesting account of mode of injury: she tripped and tumbled while cleaning her room's ceiling fan from a stool placed on a bed, and fell on the kitchen knife which was lying on the bed with some uncut vegetables. This was refuted by the police and a case of attempted suicide under Indian penal code was registered against her. Though it is not our endeavor to establish the exact mode of injury since the matter is under judicial scrutiny, still it would be prudent to note that there were no hesitation cuts or injuries in this case, moreover, the psychiatric assessment of the patient revealed no unusual personality profile with suicidal tendencies. Our medical social worker also found no social cause abetting suicide (couple was staying alone in a single room tenement away from in laws with no signs of marital discord and having a financial status befitting their economic strata in the society). Nevertheless, this clinical record underlines the importance of using simple kitchen knife with caution and restraint. In a massive internet search with Medline services/Pubmed database using the medical subject function, the authors could find no case of suicidal or accidental deep incision wound with complete cricotracheal transection and esophageal injury as defined here in. 

Some points in the early management of our patient are noteworthy and merit discussion. Cricotracheal separation can result in death. This patient survived primarily due to the prompt evacuation of the patient by the neighbours to the nearest district hospital, where the judicious tracheostomy tube insertion in the distal part of the cut trachea by the ENT surgeon secured the airway. The case thus highlights the importance of initial airway management in all such cases. Yet another factor which contributed to the survival of the patient was the immediate exploration of the injury. This not only delineated the nature of cricotracheal injury but also enabled us to detect the concomitant esophageal injury and repair it promptly. Early surgical management is also associated with significantly lower incidence of subglottic stenosis and improved outcomes with respect to airway and voice [9–11]. In addition, this clinical record also emphasizes the secondary role of high-tech investigations like CT-scan, bronchoscopy and barium swallow and so forth in diagnosis of such cases. Our experience reinforces the concept that if surgery is clearly indicated on clinical grounds, CT scan is unwarranted [12]. Moreover, critical time may be lost in procuring these investigations as patient is already in a precarious state. Thus, it is pertinent to note that in a deep incisional injury of neck, these investigations are not mandatory and surgical exploration is certainly the best diagnostic and therapeutic exercise undertaken in the best interest of patient care.

Our discussion on this case would be incomplete without addressing the controversial issue of “bilateral recurrent laryngeal nerve palsy.” A temporizing treatment in the form of tracheostomy has been provided to the patient and the patient would be reevaluated after 6 months [13]. Though spontaneous recovery of RLN has been reported in such cases associated with blunt trauma [11], we are not quite optimistic for the cut throat nature of injury in this case.

In summary, cricotracheal injury is the most immediately life-threatening laryngotracheal injury and poses significant diagnostic and therapeutic challenges. Thus, keeping our case in view, the guidelines to a successful outcome are as follows:

- quick evacuation of the patient to the nearest hospital,
- prompt airway establishment (first aid preferably by an otolaryngologist),
- immediate exploration of the incisional wound, irrespective of the zone of neck involved,
- a dogmatic approach to be avoided and each case to be treated on its own merit after assessing the injury with no room for surgical experimentation,
- investigations like CT scan, bronchoscopy and barium swallow, among others, are no substitute to surgical exploration and have no primary role.

The unusual mode of deep incisional injury by a simple kitchen knife causing cricotracheal separation with esophageal tear and its prompt management leading to survival of the patient makes this case report unique and thus prompted us to share our professional experience with the medical fraternity.
